# *Oecomys catherinae* (Sigmodontinae, Cricetidae): Evidence for chromosomal speciation?

**DOI:** 10.1371/journal.pone.0181434

**Published:** 2017-07-20

**Authors:** Stella Miranda Malcher, Julio Cesar Pieczarka, Lena Geise, Rogério Vieira Rossi, Adenilson Leão Pereira, Patricia Caroline Mary O’Brien, Paulo Henrique Asfora, Victor Fonsêca da Silva, Maria Iracilda Sampaio, Malcolm Andrew Ferguson-Smith, Cleusa Yoshiko Nagamachi

**Affiliations:** 1 Centro de Estudos Avançados da Biodiversidade, Laboratório de Citogenética, Instituto de Ciências Biológicas, Universidade Federal do Pará, Belém, Pará, Brasil; 2 Departamento de Zoologia, Laboratório de Mastozoologia, Universidade do Estado do Rio de Janeiro, Rio de Janeiro, Brasil; 3 Instituto de Biociências, Universidade Federal do Mato Grosso, Cuiabá, Mato Grosso, Brasil; 4 Cambridge Resource Center for Comparative Genomics, Department of Veterinary Medicine, University of Cambridge, Cambridge, United Kingdom; 5 Departamento de Ecologia, Instituto de Ciências Biológicas, Universidade Federal de Goiás, Goiânia, Brasil; 6 Laboratório de Genética e Biologia Molecular, Universidade Federal do Pará, Campus Universitário de Bragança, Bragança, Pará, Brasil; National Cheng Kung University, TAIWAN

## Abstract

Among the Oryzomyini (Sigmodontinae), *Oecomys* is the most speciose, with 17 species. This genus presents high karyotypic diversity (2n = 54 to 2n = 86) and many taxonomic issues at the species level because of the presence of cryptic species and the overlap of morphological characters. For these reasons the real number of species of *Oecomys* may be underestimated. With the aim of verifying if the taxon *Oecomys catherinae* is composed of more than one species, we made comparative studies on two populations from two regions of Brazil, one from the Amazon and another from the Atlantic Forest using both classical cytogenetics (G- and C-banding) and comparative genomic mapping with whole chromosome probes of *Hylaeamys megacephalus* (HME), molecular data (cytochrome b mitochondrial DNA) and morphology. Our results confirm that *Oecomys catherinae* occurs in the southeast Amazon, and reveal a new karyotype for the species (2n = 62, FNa = 62). The comparative genomic analysis with HME probes identified chromosomal homeologies between both populations and rearrangements that are responsible for the different karyotypes. We compared our results in Sigmodontinae genera with other studies that also used HME probes. These chromosomal differences together with the absence of consistent differentiation between the two populations on morphological and molecular analyses suggest that these populations may represent cryptic species.

## Introduction

Among all the genera of the Oryzomyini Tribe, *Oecomys* stands out as one of the most speciose, with 17 species currently described [1–2]. This genus is distributed in almost all Brazilian territories, but there is some uncertainty about the geographic limitation of the species because there have been no collections in some areas/biomes [3]. The taxonomy of the genus is controversial [1, 4], mainly at the species level because of the existence of cryptic species and the overlap of morphological characters as observed in other rodents [5–7]. Also, the occurrence of cryptic species in tropical ecosystems has been suggested already [8]. Bonvicino and Almeida [9] showed differences in karyotype between the species *Calomys expulsus* (2n = 66) and *C*. *callosus* (2n = 36), despite there being no statistically significant morphological differences between them. It is possible that the same is occurring in the genus *Oecomys*, as already described for *O*. *paricola* [7].

**Fig 1:**
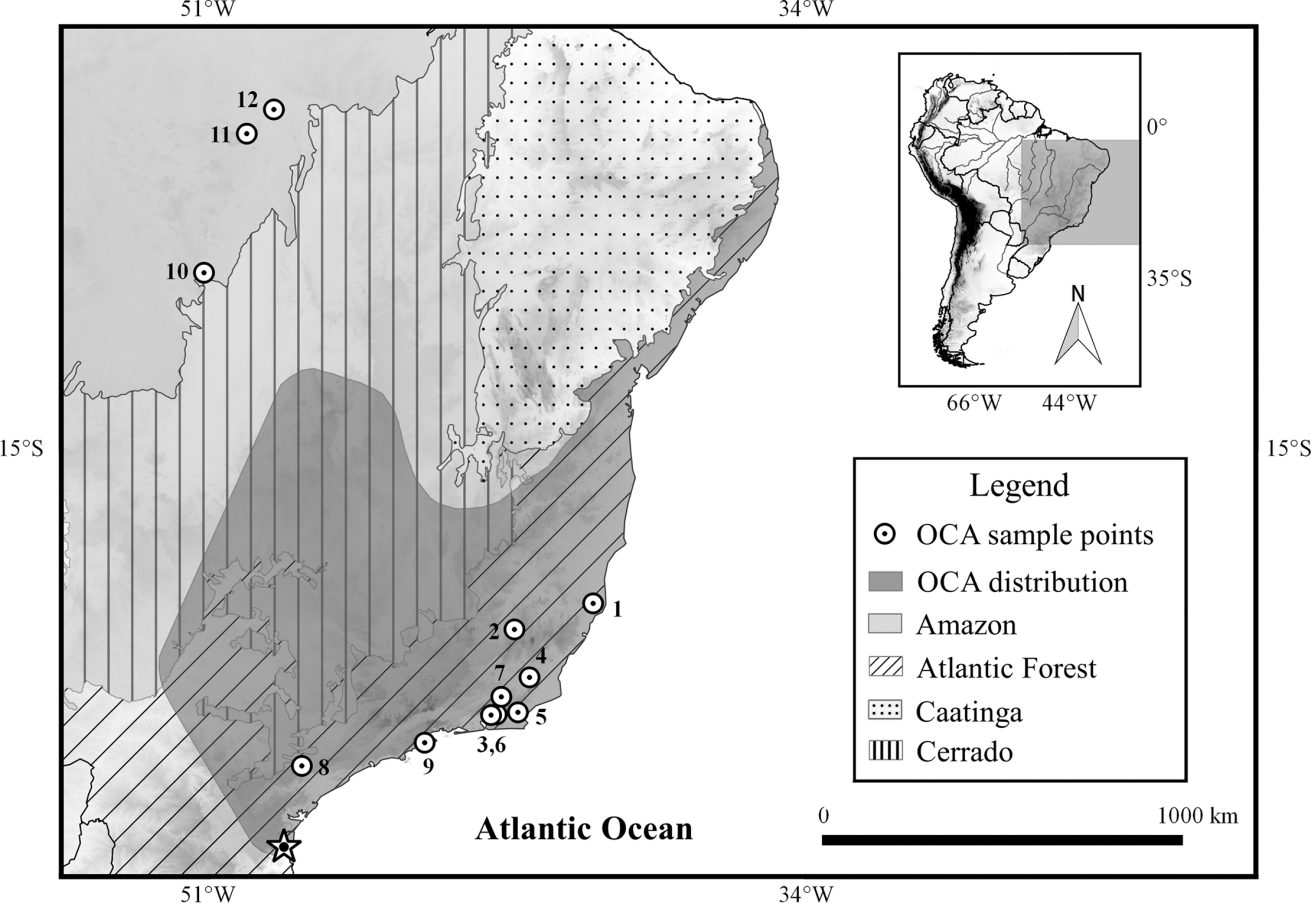
Distribution map of *Oecomys catherinae* in Brazil. Geographic distribution of *Oecomys catherinae* based on Carleton & Musser [1] and Asfora et al. [4] (shaded area) with collecting localities (circles with a dot) of specimens included in this report. 1: Linhares; 2: Pirapetinga; 3: Cachoeiras de Macacu; 4: Cambuci; 5: Casimiro de Abreu; 6: Guapimirim; 7: Sumidouro; 8: Capão Bonito; 9: Ubatuba; 10: Vila Rica; 11: Marabá; 12: Parauapebas. The star represents the type locality (Joinvile).

*Oecomys catherinae* is a Brazilian endemic species (Fig 1), typed in the Joinville municipality, in the southern state of Santa Catarina [1, 4]. This species is found throughout the Atlantic Forest, from the Santa Catarina to the Espírito Santo states, and in gallery forests in the Cerrado biome. A separate population can be found in the northern part of the Atlantic Forest, in the Alagoas, Pernambuco and Rio Grande do Norte states [4, 10–11]. Even though authors mention the occurrence of this species in the state of Bahia [11–12] and in the southeast Amazon, more precisely at the Pinkaití Research Station, southeast Pará [13–14], those reports need to be confirmed by a more accurate analysis of samples [4].

Species of the genus *Oecomys* show a high karyotypic diversity, with diploid numbers ranging from 54 chromosomes in *O*. *rutilus* to 86 chromosomes in *Oecomys* sp. [3]. Some species show the same diploid number, but with differences in the autosomal fundamental number (FNa), as in *O*. *bicolor* with 2n = 80/FNa = 116–140 [3], *O*. *superans* with 2n = 80/FNa = 108 [3, 15] and *O*. *roberti* with 2n = 80/FNa = 114 [3]. There may be cryptic species in this genus also, since Rosa et al. [7] found two different karyotypes in a sample from Belém (2n = 68/FNa = 72, 2n = 70/FNa = 76) without any morphological or molecular difference and a third karyotype in Marajó Island (2n = 70/FNa = 72) with some molecular and morphological differences.

The *Oecomys catherinae* karyotype is 2n = 60/FNa = 62–64 in individuals that occur along the coastal area of Brazil, in the Atlantic Forest, from Santa Catarina to Paraíba states and in gallery forests in the Cerrado [4, 10, 16–17]. The karyotype of 2n = 60/FNa = 62 was previously found in specimens of *O*. *concolor* from central Brazil [3, 15, 18] and in a unique specimen from Guajará-Mirim, in the state of Rondônia [19]. However, it is now known that *O*. *concolor* is restricted to the extreme northwest of Brazil, Colômbia and Venezuela [1], so the above specimens were considered to be *O*. *catherinae* because of the similarity of its karyotype [4]. The chromosomal variation and the disjunct distribution of populations identified as *O*. *catherinae* in the Atlantic Forest [4], suggest that this taxon may contain more than one species. This would suggest that the actual number of species in the genus *Oecomys* is currently underestimated.

The high chromosomal variation found in *Oecomys* brings difficulties in identifying the chromosomal rearrangements that have occurred during evolution, since classic cytogenetic banding techniques cannot identify all existing homeologies between highly rearranged karyotypes. In this context, chromosome painting has been useful in identifying chromosomal homologies [20–21] even in the highly rearranged genomes found in Sigmodontinae rodents such as *Akodon* [22–24], *Cerradomys* [25], *Necromys* [24, 26], *Oligoryzomys* [27], *Sigmodon* [28] and *Thaptomys* [26, 29]. Whole chromosome probes from *Hylaeamys megacephalus* were generated earlier [25] and have been used successfully to identify chromosomal homologies in Sigmodontinae species [24, 25, 29]. As they belong to the same subfamily, their genetic similarity allows precise hybridization of probes onto chromosomes, and the production of reliable homology maps of the species.

The present study aimed to investigate if *O*. *catherinae* represents more than one species. For this, both classical cytogenetics (G- and C-banding) and comparative genomic mapping using whole chromosome probes of *H*. *megacephalus* [25] were used in addition to molecular analysis (mitochondrial cytochrome b DNA) and morphological studies.

## Material and methods

### Ethics statement

Animals collected during this study were handled following procedures recommended by the American Society of Mammalogists. JCP and LG have a permanent field permit, number 13248 and 598633 from “Instituto Chico Mendes de Conservação da Biodiversidade”. The Cytogenetics Laboratory from UFPa has permit number 19/2003 from the Ministry of Environment for sample transport and permit 52/2003 for using the samples for research. The Ethics Committee (Comitê de Ética Animal da Universidade Federal do Pará) approved this research (Permit 68/2015). The rodents were maintained in the laboratory for 48 hours at most, free from stress in suitable rat cages, since OCA are a little smaller than rats. *Oecomys* is granivorous and frugivorous and they were feed with seeds and regional fruits bought in the city markets. Animals received food and water *ad libitum*. The room temperature was regulated to 25ºC and the light cycle was the usual day and night period of time since this is a region close to the Equator line and there is no variation on this cycle all around the year. The animals were euthanized by intraperitoneal injection of barbiturate (Pentobarbital, 120 mg/kg) after local anesthetic (lidocaine used topically).

### Sample

Twenty-eight *Oecomys catherinae* individuals were studied, from which eight were karyotyped, 23 were included in the molecular phylogenetic analysis and 16 were used in the morphological analysis. All showed the characteristics described originally for the species by Thomas [30] (S1 File). Fig 1 shows the locations where the specimens were collected. More details are provided in S1 Table and S1 File.

### Karyotype analysis

Chromosomal preparations were obtained from bone marrow of five specimens (three males and two females) collected in the municipality of Parauapebas, Pará (05°21′54″S; 49°07′24″W) and three specimens collected in the state of Rio de Janeiro, one male from the Estação Ecológica do Paraíso, Cachoeiras de Macacu municipality (22°32'35''S; 42°48'19''W), and a male and a female from the Fazenda Samburá, Cambuci municipality (21^o^29’35”S; 41^o^52’20”W) (Fig 1, S1 Table and Table 1). Chromosomal preparations of specimens from Pará were made according to Ford & Hamerton [31] and those from Rio de Janeiro were obtained from bone marrow short time culture. Cell suspensions were cultured for two hours at 37ºC in culture medium with RPMI 1640 supplemented by 20% bovine fetal serum, ethidium bromide (0.01 ml/ml in the culture medium) and colchicine 10^-6^M. After receiving a hypotonic shock for 40 min in KCl, cells in suspension were fixed in Carnoy (three parts of methanol and one of acetic acid). Staining was carried out with Giemsa and G- and C-banding followed Verma and Babu [32] and Sumner [33], respectively. FISH with telomeric probes (All Telomere Probes, Oncor) followed the manufacturer's protocol.

**Table 1:** Samples of *Oecomys catherinae*. Diploid number (2n), Fundamental Number (FNa), sex, and collection localities of *Oecomys catherinae*.

Voucher Numbers	2n	FNa	Sex	Municipality/State	Geographic coordinate
MPEG39900	62	62	M	Parauapebas/PA	05°21'54''S; 49°07'24''W
MPEG39903	62	62	F	Parauapebas/PA	05°21'54''S; 49°07'24''W
MPEG39909	62	62	F	Parauapebas/PA	05°21'54''S; 49°07'24''W
MPEG39899	62	62	M	Parauapebas/PA	05°21'54''S; 49°07'24''W
MPEG39901	62	62	M	Parauapebas/PA	05°21'54''S; 49°07'24''W
MN79852	60	62	M	Cachoeiras de Macacu/RJ	22°32'35''S; 42°48'19''W
MN 76970	60	62	M	Cambuci/RJ	21^o^29'35''S; 41^o^52'20''W
MN 76972	60	62	F	Cambuci/RJ	21^o^29'35''S; 41^o^52'20''W

MPEM = Museu Paraense Emilio Goeldi; MN = Museu Nacional; PA = Pará, RJ = Rio de Janeiro; M = Male; F = Female.

Chromosome painting with whole chromosome probes of *H*. *megacephalus* (HME; 2n = 54/FNa = 62; [25]) followed the protocol previously described [34, 25], with adaptations. DNA probes of HME, previously labeled either with biotin-16-dUTP (Boehringer Mamnheim), fluorescein isothiocyanate (FITC)-12-dUTP (Amersham) or Cy3-dUTP, were denatured at 60°C for 15 minutes. After hybridization for 72 hours at 37°C and washing the slides (2 x formamide 50%, 2x (2xSSC), 1x (4xSSC)/Tween at 40°C), the metaphases were stained with DAPI (4’,6-diamidino-2-phenylindole), for identification of the chromosomes pairs. Images were captured using the Nis-Elements software on Nikon microscope H550S, and processed using the Adobe Photoshop CS4 program.

### Molecular analysis

We used 25 *O*. *catherinae* tissue samples from the Atlantic Forest and Amazonia, of which 23 were produced in the present study. DNA was extracted using phenol-chloroform and proteinase K-RNAse protocol [35]. Partial cytochrome b (Cytb) sequences were amplified with polymerase chain reaction (PCR) with primers MVZ05 5’-CGAAGCTTGATATGAAAAACCATCGTTG-3’ and MVZ16 5’AAATAGGAARTATCAYTCTGGTTTRAT-3’ [36]. The amplification protocol consisted of initial denaturation at 94°C for 3 minutes, followed by 35 cycles of 30 seconds of denaturation at 94°C, 1 minute of annealing at 45°C and 2 minutes of extension at 72°C, with a final extension at 72°C by 7 minutes.

Sequences were edited using BioEdit 7.0.5.2 [37] and aligned with ClustalX 2.0.9 [38], following the proposed parameters of Schneider [39], with posterior manual rectification with BioEdit. Using jModeltest version 2.1.4 [40] we found GTR+I+G with a substitution rate equal to 6, gamma distribution parameter equal to 0.6270, and invariable sites in proportion equal to 0.46 as the best model for our sequences. Bayesian analysis (BI) was conducted on MrBayes 3.2.0 [41] using the evolutionary model described above, two runs, four chains, 10 million generations and sample frequency equal to 1000. The Maximum Likelihood (ML) was estimated with Garli 2.0 [42], using the above referred evolutionary model.

We also used sequences from 11 other species of *Oecomys*, *H*. *megacephalus* and *Thomasomys andersoni*, all available in GenBank (S1 Table). The latter two were used as outgroups in the phylogenetic analyses (following Rocha et al. [43]). Nonparametric bootstrapping based on 1000 replicates was performed to calculate node support values for ML. The genetic distance was estimated by MEGA 5.2 using the Kimura 2-parameter substitution model (K2P).

### Morphological analysis

We examined external and craniodental characters of 16 specimens of *O*. *catherinae*, nine of which were from the Atlantic Forest of southeastern Brazil and seven from southeastern Amazonia (Fig 1; S1 Table). Among these specimens, 15 were considered adults because they exhibited fully erupted dentition. We extracted 12 craniodental measurements with digital calipers from the adult specimens based on Voss [44]. We used the Student t-test to compare mean craniodental dimensions between the specimens from Atlantic Forest and Amazonia. Statistical analyses were performed with the software SPSS. 13.0. for Windows, at a 5% significance level. A detailed description of the morphological analysis is provided in S1 File.

## Results

### Classical cytogenetics

**Fig 2:**
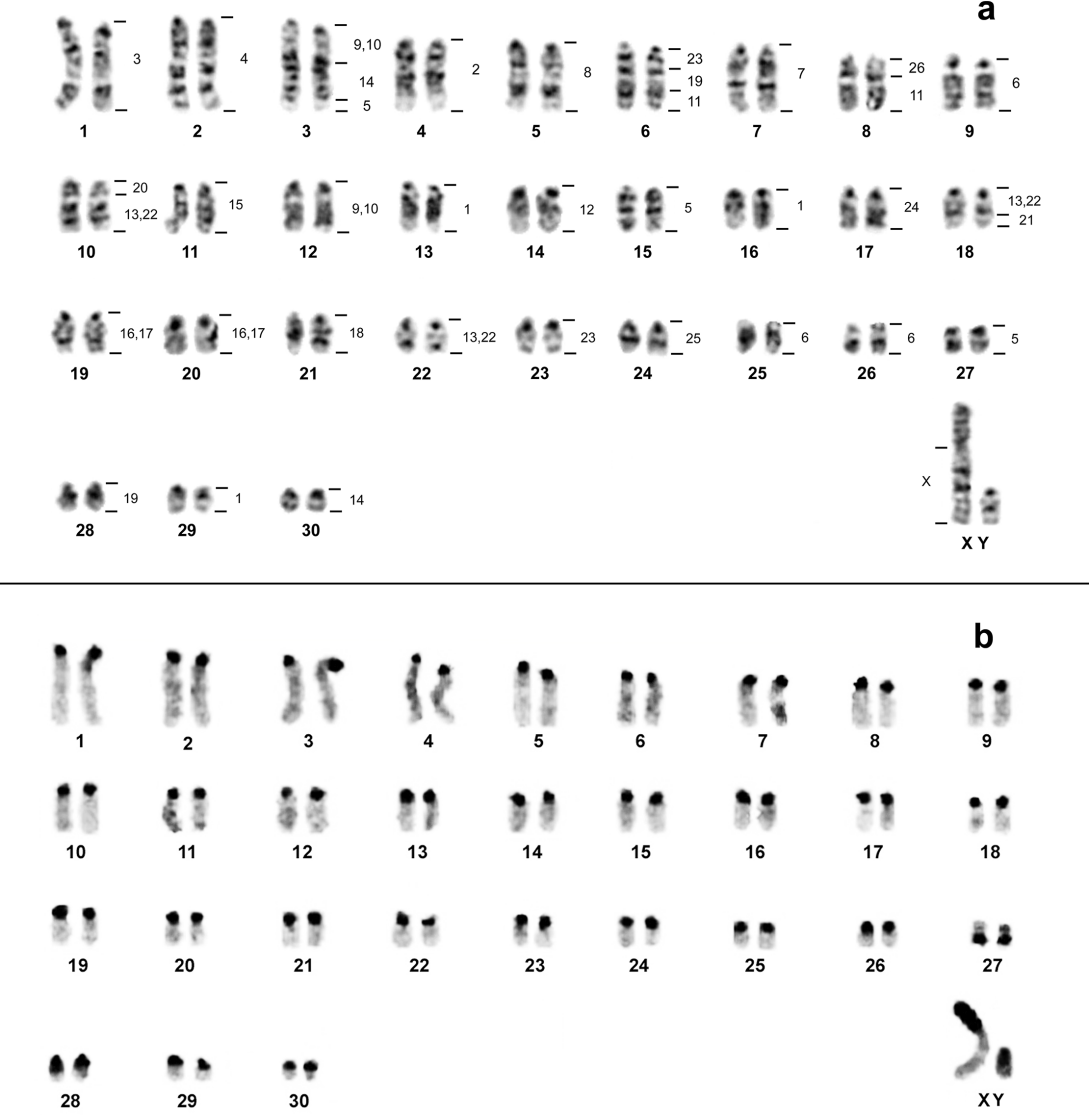
Karyotype of *O*. *catherinae* from Pará. *Oecomys catherinae* (2n = 62; FNa = 62) from the state of Pará: a) G-banding with genomic mapping using whole chromosome probes of *Hylaeamys megacephalus* (2n = 54). b) C-banding showing the Constitutive Heterochromatin location.

The diploid number of *O*. *catherinae* from Pará (OCA-PA) was 2n = 62 and FNa = 62, with a chromosomal complement of 29 pairs of acrocentric and one small pair of metacentric chromosomes (pair 27); the X chromosome is a large submetacentric and the Y is a medium sized acrocentric (Fig 2A). The Constitutive Heterochromatin (CH) is present at the pericentromeric region in all autosomal pairs; the short arm of the X chromosome is heterochromatic as is almost all the Y chromosome (Fig 2B).

**Fig 3:**
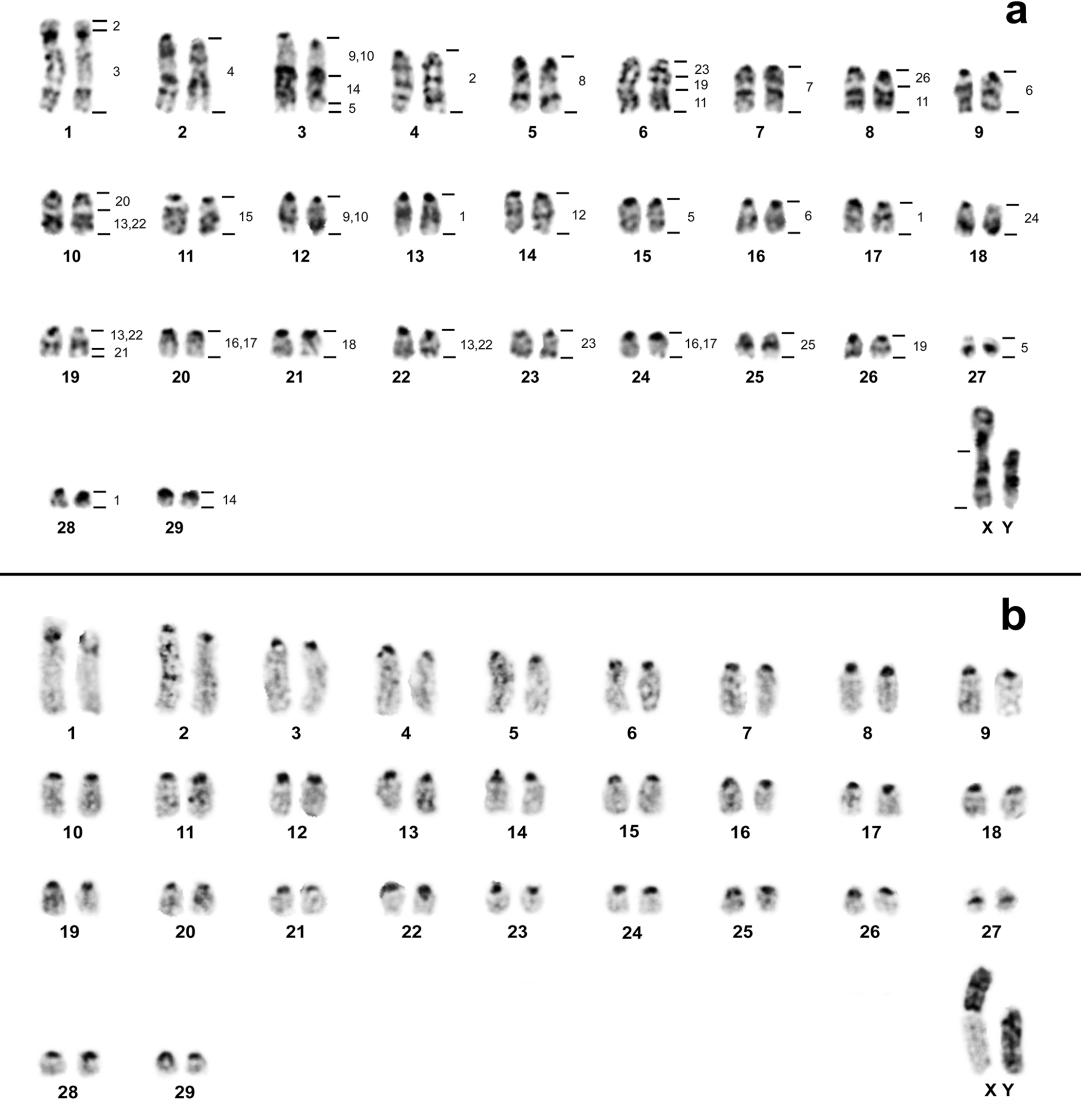
Karyotype of *O*. *catherinae* from Rio de Janeiro. *Oecomys catherinae* (2n = 60; NFa = 62) from the state of Rio de Janeiro: a) G-banding with genomic mapping using whole chromosome probes of *Hylaeamys megacephalus* (2n = 54). b) C-banding showing the Constitutive Heterochromatin location.

*Oecomys catherinae* from Rio de Janeiro (OCA-RJ) possesses 2n = 60 and FNa = 62, composed of two pairs of chromosomes with two arms (pairs 1 and 27) and 27 pairs of acrocentric chromosomes. The X chromosome is a large submetacentric and the Y is a large acrocentric (Fig 3A). The CH is present in the pericentromeric region of all autosomal pairs; the small arm of the X chromosome is all heterochromatic and the Y is almost all heterochromatic (Fig 3B).

### Molecular cytogenetics

FISH with human telomeric probes hybridized to the distal regions of all chromosomes, both in OCA-PA (S1A Fig) as in OCA-RJ (S1B Fig), but not to interstitial regions.

The results of chromosome specific probe hybridization of *H*. *megacephalus* (HME; 2n = 54 e NFa = 62, [25]) to the karyotypes of OCA-PA (2n = 62 and FNa = 62) and OCA-RJ (2n = 60 and FNa = 62) are shown in Table 2. CH regions did not show hybridization signals. In both karyotypes of OCA (PA and RJ) the 24 probes showed 38 segments of homology (Figs 2A and 3A).

**Table 2:** Number and localization of FISH signals observed in *Oecomys catherinae* from the state of Pará (OCA-PA) and *O*. *catherinae* from the state of Rio de Janeiro (OCA-RJ) with whole chromosome probes of *Hylaeamys megacephalus* (HME).

Probes of HME	Number of Signals		Chromosomal localization	
	OCA-PA	OCA-RJ	OCA-PA	OCA-RJ
1	3	3	13; 16; 29	13; 17; 28
2	1	2	4	1p; 4
3	1	1	1	1q
4	1	1	2	2
5	3	3	3q dist.; 15; 27	3q dist.; 15; 27
6	3	2	9; 25; 26	9; 16
7	1	1	7	7
8	1	1	5	5
9, 10	2	2	3q prox.; 12	3q prox.; 12
11	2	2	6q dist.; 8 dist.	6q dist.; 8q dist.
12	1	1	14	14
13, 22	3	3	10q dist.; 18q prox.; 22	10q dist.; 19q prox.; 22
14	2	2	3q int.; 30	3q int.; 29
15	1	1	11	11
16, 17	2	2	19; 20	20; 24
18	1	1	21	21
19	2	2	6q int.; 28	6q int.; 26
20	1	1	10q prox.	10q prox.
21	1	1	18q dist.	19q dist.
23	2	2	6q prox.; 23	6q prox.; 23
24	1	1	17	18
25	1	1	24	25
26	1	1	8q prox.	8q prox.
X	1	1	Xq	Xq

prox. = proximal; dist. = distal; int. = interstitial.

In OCA-PA, 14 probes of HME (2, 3, 4, 7, 8, 12, 15, 18, 20, 21, 24, 25, 26 and X) showed only one hybridization signal (in pairs 4, 1, 2, 7, 5, 14, 11, 21, 10q proximal, 18q distal, 17, 24, 8q proximal and X, respectively), from which ten (HME 2, 3, 4, 7, 8, 12, 15, 18, 24 and 25) hybridized to entire chromosomes (OCA-PA 4, 1, 2, 7, 5, 14, 11, 21, 17 and 24, respectively). Six probes (9/10, 11, 14, 16/17, 19 and 23) presented two signals, and four (1, 5, 6 and 13/22) presented three hybridization signals (Fig 2A).

In OCA-RJ, 13 probes of HME (3, 4, 7, 8, 12, 15, 18, 20, 21, 24, 25, 26 and X) showed only one hybridization signal (in pairs 1q, 2, 7, 5, 14, 11, 21, 10q proximal, 19q distal, 18, 25, 8q proximal and X, respectively), from which, eight (HME 4, 7, 8, 12, 15, 18, 24 and 25) hybridized to entire chromosomes (OCA-RJ 2, 7, 5, 14, 11, 21, 18 and 25, respectively). Eight probes (2, 6, 9/10, 11, 14, 16/17, 19 and 23) presented two signals and probes HME 1, HME 5 and HME 13/22 showed three hybridization signals (Fig 3A).

**Fig 4:**
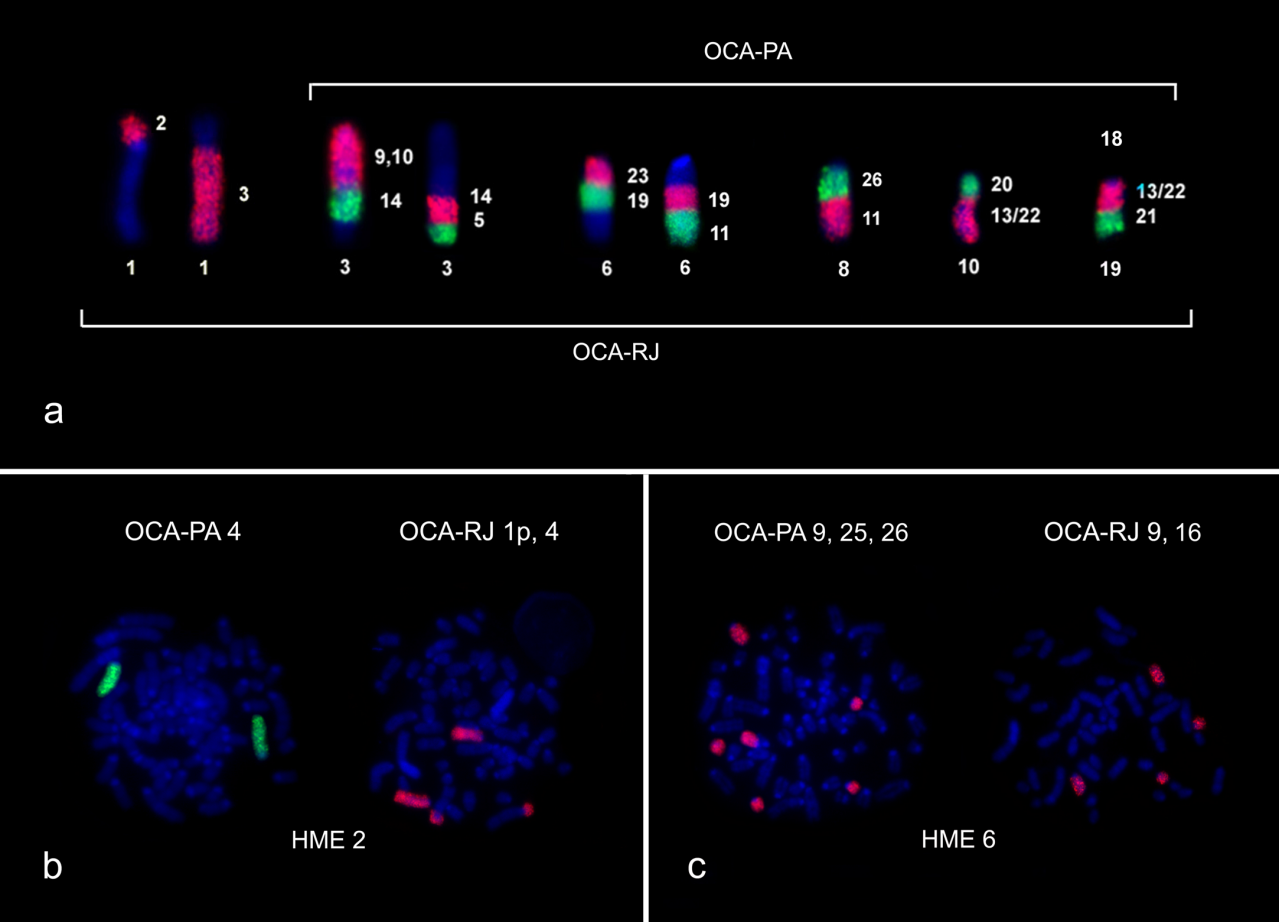
Synthetic blocks between OCA-PA and OCA-RJ. a) Syntenic association in OCA-PA and OCA-RJ; b) HME 2 painting in OCA-PA and OCA-RJ; c) HME 6 painting in OCA-PA and OCA-RJ. (DAPI = Blue; FITC = Green; Cy3 = Red).

OCA-PA has five syntenic associations: HME [9,10]/14/5 (pair 3), HME 23/19/11 (pair 6), HME 26/11 (pair 8), HME 20/[13,22] (pair 10) and HME [13,22]/21 (pair 18) (Figs 2A and 4A). OCA-RJ, in addition, has HME 2/3 (pair 1) (Figs 3A and 4A), where probe HME 2 shows only one signal in OCA-PA 4, (Figs 2A and 4B) and two signals in OCA-RJ 2 and 4 (Figs 3A and 4B). The association in OCA-RJ explains the difference in the number of chromosomes with two arms in the karyotype of OCA-RJ (pairs 1 and 27; Fig 3A) compared to the karyotype of OCA-PA (pair 27; Fig 2A). The probe HME 6 corresponds to three pairs in OCA-PA (pairs 9, 25 and 26; Figs 2A and 4C) and two in OCA-RJ (pairs 9 and 16; Figs 3A and 4C), which explains their difference in diploid number.

### Molecular phylogeny

**Fig 5:**
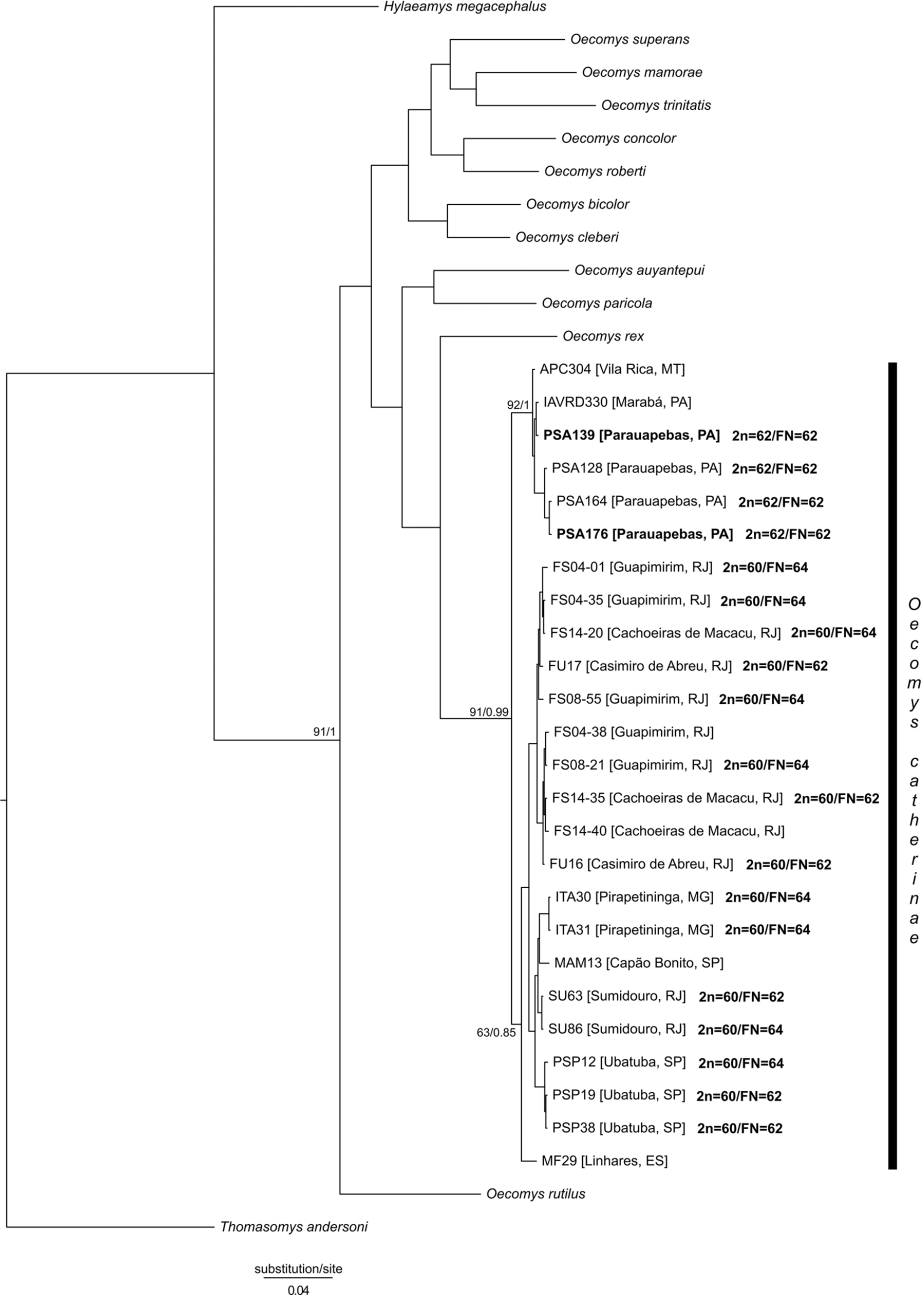
Bayesian inference tree resulted from the analyses of cytochrome b (Cytb) sequences data (Cytb). Values of bootstrap for ML / posterior probability for BI are shown close to the nodes with phylogenetic interest. For each terminal branch of *Oecomys catherinae* the alphanumeric identity and the geographic locality is provided. Bold branches refer to the samples for which karyotypic analysis was performed.

All sequences of the Cytb marker of *O*. *catherinae* resulted in 19 haplotypes and 40 polymorphic sites. The topologies obtained for ML (https://figshare.com/s/9d806a4094f4e108a764) and BI (Fig 5) indicates the monophyly of *O*. *catherinae* with high values of support (91% for ML and 0.99 for BI). Those analyses also identified two lineages within *O*. *catherinae*, one formed by individuals from the Atlantic Forest (supporting values of 63% for ML and 0.85 for BI) and another by individuals from the Amazon (supporting values of 92% for ML and 1 for BI). The Amazonian lineage includes all individuals from southern Pará and northern Mato Grosso states, while the Atlantic Forest lineage includes all individuals from the Rio de Janeiro, São Paulo, Minas Gerais and Espírito Santo states.

Among the Amazonian lineage, analyses showed the relationship with a good support only for the group formed by the samples PSA 128, PSA 164 and PSA 176, all from the locality of Parauapebas, south of Pará (93% for ML and 1 for BI). In the Atlantic forest lineage, both analyses identified the individuals from Espírito Santo as the sister group of individuals from all other localities (supporting values 63% for ML and 0.85 for BI). In this lineage, two other groups were identified indicating a geographic structure. The first group (supporting values of 68% for ML and 0.96 for BI) includes individuals from Ubatuba and Capão Bonito in the state of São Paulo, Pirapetinga in the state of Minas Gerais and Sumidouro in the state of Rio de Janeiro; the other group (supporting values 69% for ML and 0.91 for BI) includes only individuals from Guapimirim, Casimiro de Abreu and Cachoeiras de Macacu, all in the Rio de Janeiro state.

The average genetic divergence between both lineages of *O*. *catherinae* (Atlantic Forest and Amazon) was only 1.7% (SD = 0,6%), a value lower than that found between species within the genus (S2 Table). The average genetic divergence between the *O*. *catherinae* lineages and other *Oecomys* species varied from 8.5% to 12.6%, the lowest between *O*. *rex* and the Amazonian lineage of *O*. *catherinae* and the highest between *O*. *rutilus* and the Amazonian lineage of *O*. *catherinae*. The intraspecific divergence of *O*. *catherinae* was 1.5% (SD = 0.3%), with 0.3% for the Amazonian lineage and 0.8% for the Atlantic Forest lineage.

### Morphological analysis

We were not able to find any consistent differences in qualitative traits between the Atlantic Forest and the Amazonian populations of *O*. *catherinae* herein examined. In contrast, Amazonian specimens exhibited a longer incisive foramen and a wider braincase, whereas specimens from the Atlantic Forest exhibited a wider rostrum and a deeper upper incisive, with no overlapping values in this latter trait (S3 Table). A detailed description of the morphological analysis is given as supplementary material (S1 File**)**.

## Discussion

### Geographic distribution of *O*. *catherinae*

The populations of *O*. *catherinae* here studied are from the southeast Atlantic Forest, previously known as the area of occurrence of the species, and from the southeast Amazon (municipalities of Marabá and Parauapebas, state of Pará and Vila Rica, state of Mato Grosso), in a different area according to the previously known locality of the species (Fig 1). Our registers of *O*. *catherinae* in Parauapebas and Vila Rica confirm the occurrence of this species in the southeast Amazon region, as proposed by Lambert et al. [13, 14], who reported the possible occurrence of this species in the Pinkaití Research Station, in the southeast portion of the state of Pará, located 300 km from Parauapebas. In turn, Asfora et al. [4] considered a sample from Rondônia referred as *Oecomys* cf. *concolor* by Andrades-Miranda et al. [19] as *O*. *catherinae*, because of the karyotype of 2n = 60 and FNa = 62. However, this statement needs to be confirmed by morphological and molecular analyses.

### Chromosomal variation in *O*. *catherinae*

The karyotype of OCA-RJ (2n = 60, FNa = 62, Fig 3A) is similar to the one already described in the literature (for a revision see Asfora et al. [4]) and different from the karyotype with 2n = 60/FNa = 64 caused by a pericentric inversion of one small pair of acrocentric chromosomes in the karyotype with FNa = 62 to a metacentric in the karyotype with FNa = 64.

OCA-PA presents a new karyotype for the species, with 2n = 62/FNa = 62 (Fig 2A). Our comparative genomic mapping with HME probes allows the identification of all chromosomal homeologies, and rearrangements that differentiate the karyotypes. The results show that the karyotypes of OCA-RJ and OCA-PA differ by two chromosomal rearrangements, which can explain the difference in the 2n of 60 to 62 chromosomes, where HME6, which corresponds to two pairs (9 and 16; Figs 3A and 4C) in the karyotype of OCA-RJ and to three pairs (9, 25 and 26) in the karyotype of OCA-PA (Figs 2A and 4C). Possibly this difference arose from a tandem fusion or fission, where chromosomes 25 and 26 of OCA-PA correspond to chromosome 16 of OCA-RJ (Fig 4). In addition a translocation explains the difference in the number of metacentric pairs, in which HME2, homologous to pair 4 in the karyotype of OCA-PA (Figs 2A and 4B), corresponds to pair 4 of OCA-RJ plus the short arm of pair 1 (Figs 3A and 4B), that forms the HME 2/3 association (Fig 4A). Also, both karyotypes differ from each other by the size of the X and Y chromosomes, because of the difference in the amount of CH (Figs 2B and 3B). Such rearrangements may lead to problems during gametogenesis, producing gametes with low viability, and thus reduced fertility [45]. Similar results with similar conclusions were found by other authors on rodent chromosomal studies [9, 46]. The reduction in fertility between populations may result in reproductive isolation, and this may be reinforced by the probably allopatric distribution of these populations. Alternatively populations were first geographically separated and, following this separation, chromosomal divergence occurred/emerged and got fixed.

In studies of other Sigmodontinae genera using HME probes, it is possible to verify the organization of the syntenic group that corresponds to chromosomes 2 and 6 of *H*. *megacephalus* [25]. In *Cerradomys langguthi* [25], *Akodon montensis*, *Thaptomys nigrita* [29], *Necromys lasiurus* and *Akodon* sp. [24], the HME2 chromosome is divided into two syntenic blocks of similar size that correspond to chromosomes 4 and 5 of *Mus musculus* [24], the probable ancestral form. In OCA-PA these two blocks are united as in *H*. *megacephalus*, but in OCA-RJ they are fragmented in two blocks of unequal size, the smaller fragment resulting from a translocation (described previously). Thus, our results indicate that in *Oecomys* the original chromosomal form might be two united blocks (OCA-PA) and a translocation in one portion of this block to the homologous chromosome of HME3 (OCA-RJ) would be the derived form, probably an autapomorphic trait. In turn, the HME6 chromosome in those species mentioned above is present as a unique block, associated with HME21 (with the exception of *H*. *megacephalus*), corresponding to an ancestral character, the association HME6/21 and chromosome 2 of *M*. *musculus* [24]. Therefore, the syntenic groups corresponding to HME6 in *Oecomys*, both in OCA-PA as in OCA-RJ, are derivative. A more robust analysis of the genus *Oecomys* will be necessary to define with certainty which karyotype in OCA is the derived form.

In comparing the results of the morphological, molecular and cytogenetic analyses, we were able to observe significant chromosomal variation between the samples from the Amazon and the Atlantic Forest. There seems to have been absence of hybridization and introgression between both populations, meaning an absence of gene flow. While most morphological characteristics do not show consistent differences in the two populations, four craniodental measurements differ significantly between them (S3 Table). The molecular analysis (Fig 5) shows that the medium genetic divergence between populations is only 1.7% (SD = 0.6%), a value lower that those observed between species within the same genus (8.5% to 12.6%) [15]. These chromosomal differences described here, in association with the absence of consistent differentiation between the two populations in our molecular analysis, suggest that these populations could be cryptic species, in which craniodental differences could indicate the beginnings of morphological differentiation.

## Conclusion

The present paper confirms the presence of *O*. *catherinae* in the Amazonian biome, enlarging its geographic distribution in South America. These populations also contain a new karyotype for the species, with 2n = 62/FNa = 62. Comparative genomic mapping shows that this karyotype differs from all others already published, by fission-fusion and translocation. However, these disjunct populations from the Amazon and Atlantic Forest may represent two cryptic species, with chromosomal data and geographic distance indicating the absence of gene flow between them, although morphological and molecular data do not show significant differences. Our data indicate that further studies and more fieldwork for increasing samples are welcome to improve knowledge about the diversity of this small mammal in the Amazon and Atlantic Forest.

## Supporting information

S1 File Methods and results of the morphological analysis of the sample. (DOCX)

S1 Table List of specimens included in the present study. For each specimen, voucher and/or field number, GenBank accession number, locality, and type of analysis are provided (Cyt = Cytogenetics; Mol = Molecular; Morph = Morphology). In bold, sequences produced in the present study. States are ES (Espírito Santo), MG (Minas Gerais), MT (Mato Grosso), PA (Pará), RJ (Rio de Janeiro), and SP (São Paulo). Localities are plotted on map (Fig 1).
(DOCX)

S2 Table Estimates of evolutionary divergence over sequence pairs between groups. The numbers of base substitutions per site from averaging over all sequence pairs between groups are shown. Standard error estimate(s) are shown above the diagonal. Analyses were conducted using the Kimura 2-parameter model [1]. The rate variation among sites was modeled with a gamma distribution (shape parameter = 0.627). The analysis involved 38 nucleotide sequences. Codon positions included were 1st+2nd+3rd+Noncoding. All positions containing gaps and missing data were eliminated. There were a total of 352 positions in the final dataset. Evolutionary analyses were conducted in MEGA6 [2].
(DOCX)

S3 Table Descriptive statistics of *Oecomys catherinae* populations from the Atlantic Forest and Amazon analyzed in the present study. Values correspond to average±standar-deviation / min.-max. * Variables with 0.05 > p > 0.01 for the Student t test. ** Variables with p < 0.01 for the Student t test. Craniodental dimensions follow Voss [1] as follows: **CIL** = condyle-incisive length; **LD** = length of diastema; **LM** = length of molars; **BM1** = breadth of M1; **LIF** = length of incisive foramen; **BR** = breadth of rostrum; **BPB** = breadth of palatal bridge; **BZP** = breadth of zygomatic plate; **LIB** = least interorbital breadth; **BB =** breadth of braincase; **DI =** depth of incisor; and **LOF =** length of orbital fossa.
(DOCX)

S1 Fig *In situ* fluorescent hybridization (FISH) in *Oecomys catherinae* with telomeric probes. a) In OCA-PA (green). b) In OCA-RJ (red).
(TIF)
