# Supplementary material for: Oecomys catherinae (Sigmodontinae, Cricetidae): Evidence for chromosomal speciation?
Source: PLoS One. 2017 Jul 20;12(7):e0181434. doi: 10.1371/journal.pone.0181434 (PMC5519095; doi:10.1371/journal.pone.0181434)
Supplement: S1 File — (DOCX) [file pone.0181434.s001.docx]

**Morphological Analysis of the Sample**

**Methods**

The specimens were assigned to *Oecomys catherinae* from the following characters that match Thomas's [29] original description of the species: large size, dorsal hairs about 13 mm in length, dorsal pelage reddish, ventral pelage composed of gray-based hairs, brownish patch on metatarsals, no conspicuous caudal tuft, supraorbital ridges very developed and passing back as strongly marked ridges across the parietals, and zygomatic plate anteriorly projected, turning the zygomatic notch notable at dorsal view.

We examined external and craniodental characters of 16 specimens of *O. catherinae*, nine from the Atlantic Forest of southeastern Brazil and seven from southeastern Amazonia (Fig. 1; S1 Table). Among these specimens, 15 were considered adults because they exhibited fully erupted dentition. We extracted 12 craniodental measurements with digital calipers from the adult specimens based on Voss [1], as follows: CIL, condyle-incisive length (measured from the greater curvature of an upper incisor to the articular surface of the occipital condyle on the same side); LD, length of diastema (measured from the crown of the first maxillary molar to the exposed lesser curvature of the upper incisor on the same side); LM, length of molars (occlusal length of the maxillary molar row); BM1, breadth of M 1 (breadth of the first molar across the protocone-paracone cusp pair); LIF, length of incisive foramen (inside length of one incisive foramen); BR, breadth of rostrum (measured just inside the antero-ventral edge of the zygomatic plate); BPB, breadth of palatal bridge (measured between the protocones of the first maxillary molars); BZP, breadth of zygomatic plate (least distance between anterior and posterior edges of the zygomatic plate); LIB, least interorbital breadth (least distance across the frontal bones between the orbital fossae); BB, breadth of braincase (measured immediately dorsal to the squamosal roots of the zygomatic arches and ventral to the temporal ridges); DI, depth of incisor (distance between greater and lesser curvatures of an upper tooth); and LOF, length of orbital fossa (greatest dimension of the orbital fossa inside the maxillary and squamosal roots of the zygomatic arch). We used Student’s t test to compare means of craniodental dimensions between the specimens of Atlantic Forest and Amazonia. Statistical analyses were performed with the software SPSS. 13.0. for Windows, considering a significance level of 5 %.

**Results**

We were not able to find any consistent qualitative traits that consistently discriminate the Atlantic Forest and the Amazonian populations of *O. catherinae* herein examined. Specimens of both populations (except when otherwise mentioned) exhibited dorsal pelage soft, varying from light orange-grayish to red-grayish; dorsal hairs 11-13 mm in length; ventral pelage totally gray-based and yellowish buffy-tipped, usually with a thin or small pure yellowish white spot on throat in specimens from Amazonia; the larger mystacial vibrissa surpasses the ears when laid back against the head; tail dark brown, with the ventral proximal part lighter; all three caudal scale hairs similarly thick, with the central hair slightly longer, reaching two scales rows in length; caudal tuft absent; feet and hands usually pure buffy colored with a light brownish spot on metatarsals, with squamae in plantar surface and dermal fold in fingers; ungual tufts as long as or slightly longer than claws. Craniodentally, they exhibited supraorbital ridges well developed, passing back as strongly marked ridges across the parietals; broad zygomatic plate, with small projection of the anterior edge, turning the zygomatic notch notable at dorsal view; continuous frontal-parietal and frontal-squamosal sutures; parietal bones slightly expanded below the lateral surface of the braincase; interparietal bone a little broader than longer in the Amazonian species, while it is as broad as long in the specimens from the Atlantic Forest resulting in a more or less rounded interparietal; one or two large posterolateral pits on palate; incisive foramina usually long and narrow, but never reaching the line of M1; anterior edge of mesopterygoid fossa slightly rounded or squared, usually not surpassing the posterior edge of the maxillary bone; roof of mesopterygoid fossa totally ossified in the Amazonian specimens from Amazonia, but variably ossified in specimens from Atlantic Forest; subsquamosal process very short and broad, leading to a small subsquamosal fenestra; alisphenoid strut absent at both sides; anterior opening of the alisphenoid canal large and present at both sides; pattern 1 of carotid and stapedial circulatory system described by Voss [2]; mental foramen opening frontally; both lower and upper masseteric ridges conjoined as a single crest under m1 alveoli; capsular process of lower incisor alveoli well developed; anteroflexus as a single internal fossette on M1; protoflexus absent on M2; accessory loph posterior to the paracone usually present in the specimens from Atlantic Forest, but variably present in the Amazonian specimens; mesoflexus as a single internal fossette on M2; hypoflexus present on M3, shallow in the specimens from the Atlantic Forest, but variably shallow in the Amazonian specimens; and deep posteroflexid present on m3.

In contrast, Amazonian specimens exhibited significantly longer incisive foramen (variable LIF) and wider braincase (variable BB), whereas specimens from the Atlantic Forest exhibited significantly wider rostrum (variable BR) and deeper upper incisive (variable DI), with no overlapping values in this latter trait (S3 Table).

**References**

1. Voss RS. An introduction to the Neotropical muroid rodent genus *Zygodontomys*. Bulletin of the American Museum of Natural History. 1991; 210, pp. 1–113.

2. Voss RS. Systematics and ecology of ichthyomyine rodents (Muroidea): patterns of morphological evolution in a small adaptive radiation. Bulletin of the American Museum of Natural History. 1988; 188: 259-493.
