# Supplementary material for: Oecomys catherinae (Sigmodontinae, Cricetidae): Evidence for chromosomal speciation?
Source: PLoS One. 2017 Jul 20;12(7):e0181434. doi: 10.1371/journal.pone.0181434 (PMC5519095; doi:10.1371/journal.pone.0181434)
Supplement: S1 Table — For each specimen, voucher and/or field number, GenBank accession number, locality, and type of analysis are provided (Cyt = Cytogenetics; Mol = Molecular; Morph = Morphology). In bold, sequences produced in the present study. States are ES (Espírito Santo), MG (Minas Gerais), MT (Mato Grosso), PA (Pará), RJ (Rio de Janeiro), and SP (São Paulo). Localities are plotted on map (Fig 1). (DOCX) [file pone.0181434.s002.docx]

**S1 Table. List of specimens included in the present study**. For each specimen, voucher and/or field number, GenBank accession number, locality, and type of analysis are provided (Cyt = Cytogenetics; Mol = Molecular; Morph = Morphology). In bold, sequences produced in the present study. States are ES (Espírito Santo), MG (Minas Gerais), MT (Mato Grosso), PA (Pará), RJ (Rio de Janeiro), and SP (São Paulo). Localities are plotted on map (Fig. 1).

| **Species** | **Voucher Number** | **Field Number** | **GenBank Accession Number** | **Locality** | **Cyt** | **2n** | **Mol** | **Morph** |
| --- | --- | --- | --- | --- | --- | --- | --- | --- |
| *Oecomys catherinae* | -- | MF29 | EU579507 | ES: Linhares | -- | -- | X | -- |
| *Oecomys catherinae* | MN72737 | ITA30 | KY605398 | MG: Pirapitinga | -- | 60* | X | -- |
| *Oecomys catherinae* | MN72738 | ITA31 | KY605399 | MG: Pirapitinga | -- | 60* | X | -- |
| *Oecomys catherinae* | MN74371 | FS14-20 | KY605393 | RJ: Cachoeiras de Macacu | -- | 60 [1] | X | Skull |
| *Oecomys catherinae* | NM74372 | FS14-35 | KY605394 | RJ: Cachoeiras de Macacu | -- | 60 [1] | X | Skin, skull |
| *Oecomys catherinae* | MN79852 | MFD01 | -- | RJ: Cachoeiras de Macacu | X | 60 ** | -- | -- |
| *Oecomys catherinae* | MN76970 | RB-M-3-51 | -- | RJ: Cambuci | X | 60 ** | -- | -- |
| *Oecomys catherinae* | MN76972 | RB-M-3-52 | -- | RJ: Cambuci | X | 60 ** | -- | -- |
| *Oecomys catherinae* | -- | FU16 | KY605396 | RJ: Casimiro de Abreu | -- | 60 [1] | X | -- |
| *Oecomys catherinae* | -- | FU17 | KY605397 | RJ: Casimiro de Abreu | -- | 60 [1] | X | -- |
| *Oecomys catherinae* | MN74359 | FS04-01 | KY605388 | RJ: Guapimirim | -- | 60 [1] | X | Skin, skull |
| *Oecomys catherinae* | MN74360 | FS04-35 | KY605389 | RJ: Guapimirim | -- | 60 [1] | X | Skin, skull |
| *Oecomys catherinae* | MN74366 | FS08-21 | KY605391 | RJ: Guapimirim | -- | 60 [1] | X | Skull |
| *Oecomys catherinae* | MN74368 | FS08-55 | KY605392 | RJ: Guapimirim | -- | 60 [1] | X | Skin, skull |
| *Oecomys catherinae* | -- | SU63 | KY605403 | RJ: Sumidouro | -- | 60 [1] | X | -- |
| *Oecomys catherinae* | -- | SU86 | KY605404 | RJ: Sumidouro | -- | 60 [1] | X | -- |
| *Oecomys catherinae* | MVZ200982 | MAM13 | HM594616 | SP: Capão Bonito | -- | -- | X | -- |
| *Oecomys catherinae* | MN74377 | PSP12 | KY605400 | SP: Ubatuba | -- | 60 [2] | X | Skin, skull |
| *Oecomys catherinae* | MN74378 | PSP19 | KY605401 | SP: Ubatuba | -- | 60 [2] | X | -- |
| *Oecomys catherinae* | MN74379 | PSP38 | KY605402 | SP: Ubatuba | -- | 60 [2] | X | -- |
| *Oecomys catherinae* | MZUSP29533 | APC304 | KY605382 | MT: Vila Rica | -- | -- | X | -- |
| *Oecomys catherinae* | MPEG38898 | IAVRD330 | KY605383 | PA: Marabá | -- | -- | X | Skin, skull |
| *Oecomys catherinae* | MPEG38976 | IAVRD1169 | -- | PA: Marabá | -- | -- | -- | -- |
| *Oecomys catherinae* | MPEG39900 | PSA139 | KY605385 | PA: Parauapebas | X | 62 ** | X | Skin, skull |
| *Oecomys catherinae* | MPEG39903 | PSA176 | KY605387 | PA: Parauapebas | X | 62 ** | X | Skin, skull |
| *Oecomys catherinae* | MPEG39899 | PSA128 | KY605384 | PA: Parauapebas | X | 62 ** | X | Skin, skull |
| *Oecomys catherinae* | MPEG39901 | PSA164 | KY605386 | PA: Parauapebas | X | 62 ** | X | Skin, skull |
| *Oecomys catherinae* | MPEG39909 | PSA205 | -- | PA: Parauapebas | X | 62 ** | -- | Skin, skull |
| *Oecomys auyantepui* | -- | -- | AJ496304 | -- | -- | -- | X | -- |
| *Oecomys bicolor* | MVZ154997 | -- | JQ312124 | -- | -- | -- | X | -- |
| *Oecomys cleberi* | -- | LPC466 | HM594613 | -- | -- | -- | X | -- |
| *Oecomys concolor* | MVZ155005 | -- | JF693876 | -- | -- | -- | X | -- |
| *Oecomys mamorae* | -- | LPC603 | HM594606 | -- | -- | -- | X | -- |
| *Oecomys paricola* | -- | PAB032 | JF759681 | -- | -- | -- | X | -- |
| *Oecomys rex* | -- | -- | AJ496314 | -- | -- | -- | X | -- |
| *Oecomys roberti* | -- | RGR379 | HM594604 | -- | -- | -- | X | -- |
| *Oecomys rutilus* | -- | -- | AJ496313 | -- | -- | -- | X | -- |
| *Oecomys superans* | -- | -- | AY275123 | -- | -- | -- | X | -- |
| *Oecomys trinitatis* | -- | -- | GU126527 | -- | -- | -- | X | -- |
| *Hylaeamys megacephalus* | MHNLS8061 | -- | EU579499 | -- | -- | -- | X | -- |
| *Thomasomys andersoni* | AMNH268734 | -- | DQ914644 | -- | -- | -- | X | -- |

(*) Lena Geise - Personal communication.

(**) Present study

1. Asfora PH, Palma ART, Astúa D and Geise L. Distribution of *Oecomys catherinae* Tomas, 1909 (Rodentia: Cricetidae) in northeastern Brazil with karyotypical and morphometrical notes. Biota Neotrop. 2011;11 (2): 415-424. doi: 10.1590/S1676-06032011000200039.

2. Pinheiro OS, Geise L. Non-volant mammals of Picinguaba, Ubatuba, state of São Paulo, southeastern Brazil. Bol. Mus. Biol. Mello Leitão. 2008;23: 51-59.
