# Supplementary material for: Oecomys catherinae (Sigmodontinae, Cricetidae): Evidence for chromosomal speciation?
Source: PLoS One. 2017 Jul 20;12(7):e0181434. doi: 10.1371/journal.pone.0181434 (PMC5519095; doi:10.1371/journal.pone.0181434)
Supplement: S2 Table — The numbers of base substitutions per site from averaging over all sequence pairs between groups are shown. Standard error estimate(s) are shown above the diagonal. Analyses were conducted using the Kimura 2-parameter model [1]. The rate variation among sites was modeled with a gamma distribution (shape parameter = 0.627). The analysis involved 38 nucleotide sequences. Codon positions included were 1st+2nd+3rd+Noncoding. All positions containing gaps and missing data were eliminated. There were a total of 352 positions in the final dataset. Evolutionary analyses were conducted in MEGA6 [2]. (DOCX) [file pone.0181434.s003.docx]

**S2 Table.** **Estimates of evolutionary divergence over sequence pairs between groups.** The number of base substitutions per site from averaging over all sequence pairs between groups are shown. Standard error estimate(s) are shown above the diagonal. Analyses were conducted using the Kimura 2-parameter model [1]. The rate variation among sites was modeled with a gamma distribution (shape parameter = 0.627). The analysis involved 38 nucleotide sequences. Codon positions included were 1st+2nd+3rd+Noncoding. All positions containing gaps and missing data were eliminated. There were a total of 352 positions in the final dataset. Evolutionary analyses were conducted in MEGA6 [2].

| **Taxa** | **1** | **2** | **3** | **4** | **5** | **6** | **7** | **8** | **9** | **10** | **11** | **12** | **13** | **14** | **15** |
| --- | --- | --- | --- | --- | --- | --- | --- | --- | --- | --- | --- | --- | --- | --- | --- |
| 1. *H. megacephalus* |  | 0.053 | 0.044 | 0.044 | 0.036 | 0.033 | 0.040 | 0.039 | 0.035 | 0.041 | 0.037 | 0.037 | 0.035 | 0.037 | 0.034 |
| 2. *T. andersoni* | 0.299 |  | 0.043 | 0.043 | 0.046 | 0.052 | 0.052 | 0.042 | 0.044 | 0.054 | 0.051 | 0.041 | 0.045 | 0.046 | 0.049 |
| 3. *O. superans* | 0.241 | 0.265 |  | 0.019 | 0.024 | 0.025 | 0.022 | 0.020 | 0.021 | 0.022 | 0.022 | 0.019 | 0.021 | 0.021 | 0.023 |
| 4. *O. concolor* | 0.248 | 0.258 | 0.088 |  | 0.029 | 0.026 | 0.022 | 0.024 | 0.017 | 0.021 | 0.022 | 0.020 | 0.026 | 0.020 | 0.019 |
| 5. *O. rutilus* | 0.203 | 0.276 | 0.124 | 0.162 |  | 0.024 | 0.024 | 0.026 | 0.025 | 0.027 | 0.028 | 0.028 | 0.026 | 0.025 | 0.024 |
| 6. *O. auyantepui* | 0.177 | 0.297 | 0.122 | 0.132 | 0.116 |  | 0.024 | 0.022 | 0.024 | 0.028 | 0.027 | 0.025 | 0.021 | 0.022 | 0.020 |
| 7. *O. bicolor* | 0.217 | 0.309 | 0.107 | 0.111 | 0.121 | 0.121 |  | 0.025 | 0.025 | 0.020 | 0.016 | 0.023 | 0.024 | 0.022 | 0.020 |
| 8. *O. paricola* | 0.211 | 0.258 | 0.098 | 0.124 | 0.138 | 0.103 | 0.118 |  | 0.022 | 0.027 | 0.025 | 0.023 | 0.023 | 0.021 | 0.021 |
| 9. *O. roberti* | 0.185 | 0.257 | 0.096 | 0.072 | 0.124 | 0.111 | 0.116 | 0.111 |  | 0.019 | 0.019 | 0.019 | 0.025 | 0.024 | 0.022 |
| 10. *O. mamorae* | 0.222 | 0.323 | 0.104 | 0.103 | 0.138 | 0.141 | 0.094 | 0.138 | 0.080 |  | 0.020 | 0.023 | 0.030 | 0.025 | 0.024 |
| 11. *O. cleberi* | 0.200 | 0.304 | 0.107 | 0.111 | 0.141 | 0.131 | 0.062 | 0.128 | 0.091 | 0.091 |  | 0.020 | 0.028 | 0.025 | 0.023 |
| 12. *O. trinitatis* | 0.200 | 0.248 | 0.082 | 0.094 | 0.151 | 0.122 | 0.110 | 0.122 | 0.080 | 0.104 | 0.098 |  | 0.021 | 0.021 | 0.022 |
| 13. *O. rex* | 0.204 | 0.274 | 0.099 | 0.131 | 0.136 | 0.104 | 0.124 | 0.115 | 0.126 | 0.153 | 0.148 | 0.105 |  | 0.018 | 0.020 |
| 14. *O. catheriane* (Amazônia) | 0.202 | 0.287 | 0.103 | 0.094 | 0.126 | 0.102 | 0.110 | 0.098 | 0.114 | 0.123 | 0.122 | 0.108 | 0.085 |  | 0.006 |
| 15. *O. catherinae* (Mata Atlântica) | 0.194 | 0.300 | 0.107 | 0.090 | 0.125 | 0.095 | 0.099 | 0.102 | 0.107 | 0.120 | 0.113 | 0.110 | 0.093 | 0.017 |  |

**1.** Kimura M. A simple method for estimating evolutionary rate of base substitutions through comparative studies of nucleotide sequences. J Mol Evol. 1980; 16: 111-120.

**2.** Tamura K, Stecher G, Peterson D, Filipski A, and Kumar S. MEGA6: Molecular Evolutionary Genetics Analysis version 6.0. Mol Biol Evol. 2013; 30: 2725-2729.
