# Supplementary material for: Oecomys catherinae (Sigmodontinae, Cricetidae): Evidence for chromosomal speciation?
Source: PLoS One. 2017 Jul 20;12(7):e0181434. doi: 10.1371/journal.pone.0181434 (PMC5519095; doi:10.1371/journal.pone.0181434)
Supplement: S3 Table — Values correspond to average±standar-deviation / min.-max. * Variables with 0.05 > p > 0.01 for the Student t test. ** Variables with p < 0.01 for the Student t test. Craniodental dimensions follow Voss [1] as follows: CIL = condyle-incisive length; LD = length of diastema; LM = length of molars; BM1 = breadth of M1; LIF = length of incisive foramen; BR = breadth of rostrum; BPB = breadth of palatal bridge; BZP = breadth of zygomatic plate; LIB = least interorbital breadth; BB = breadth of braincase; DI = depth of incisor; and LOF = length of orbital fossa. (DOCX) [file pone.0181434.s004.docx]

**S3 Table:** Descriptive statistics of *Oecomys catherinae* populations from the Atlantic Forest and Amazon analyzed in the present study.

| **Variable** | ***O. catherinae***  **Atlantic Forest (n=8)** | ***O. catherinae***  **Amazon (n=6)** |
| --- | --- | --- |
| CIL | 30,39±1,64  27,86-32,68 | 30,34±1,07  29,34-32,33 |
| LD | 8,43±0,62  7,58-9,03 | 8,47±0,52  7,92-9,32 |
| LM | 5,13±0,19  4,92-5,5 | 5,07±0,18  4,81-5,29 |
| BM1 | 1,43±0,09  1,34-1,62 | 1,45±0,08  1,32-1,55 |
| LIF * | 5,01±0,48  4,31-5,72 | 5,54±0,24  5,14-5,81 |
| BR ** | 5,72±0,39  5,22-6,32 | 4,99±0,34  4,40-5,32 |
| BPB | 3,2±0,25  2,87-3,49 | 3,23±0,22  2,92-3,50 |
| BZP | 3,83±0,32  3,31-4,40 | 3,53±0,14  3,36-3,77 |
| LIB | 5,96±0,35  5,58-6,50 | 5,76±0,49  5,32-6,51 |
| BB ** | 12,91±0,49  12,39-13,69 | 13,67±0,23  13,38-14,05 |
| DI ** | 2,63±0,21  2,32-2,95 | 1,92±0,11  1,83-2,07 |
| LOF | 11,58±0,48  10,69-12,08 | 11,83±0,39  11,28-12,32 |

Values correspond to average±standar-deviation / min.-max. * Variables with 0.05 > p > 0.01 for the Student t test. ** Variables with p < 0.01 for the Student t test. Craniodental dimensions follow Voss [1] as follows: **CIL** = condyle-incisive length; **LD** = length of diastema; **LM** = length of molars; **BM1** = breadth of M1; **LIF** = length of incisive foramen; **BR** = breadth of rostrum; **BPB** = breadth of palatal bridge; **BZP** = breadth of zygomatic plate**; LIB** = least interorbital breadth; **BB =** breadth of braincase; **DI =** depth of incisor; and **LOF =** length of orbital fossa.

1. Voss RS. An introduction to the Neotropical muroid rodent genus *Zygodontomys*. Bulletin of the American Museum of Natural History. 1991; 210, pp. 1–113.
