# Supplementary figures and images for: Oecomys catherinae (Sigmodontinae, Cricetidae): Evidence for chromosomal speciation?
Source: PLoS One. 2017 Jul 20;12(7):e0181434. doi: 10.1371/journal.pone.0181434 (PMC5519095; doi:10.1371/journal.pone.0181434)

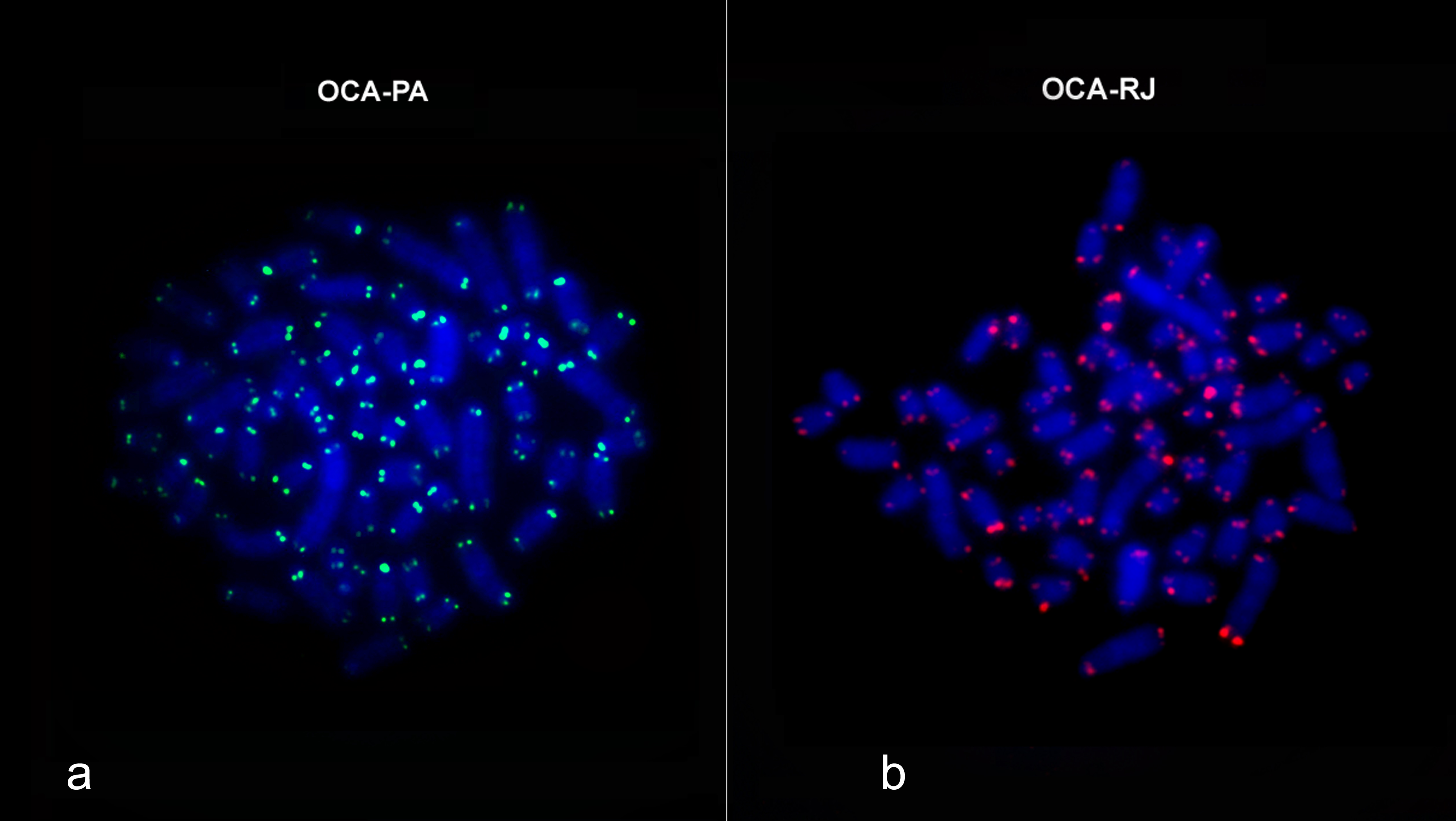

Supplement: S1 Fig — a) In OCA-PA (green). b) In OCA-RJ (red). (TIF) [file pone.0181434.s005.tif]
